# Carfilzomib‐Induced Thrombotic Microangiopathy—Two Case Reports

**DOI:** 10.1002/cnr2.2163

**Published:** 2024-10-10

**Authors:** Irene Attucci, Sofia Pilerci, Maria Messeri, Ludovica Pengue, Giulia Tomasino, Leonardo Caroti, Alessandro M. Vannucchi, Elisabetta Antonioli

**Affiliations:** ^1^ Haematology Unit Careggi University Hospital Florence Italy; ^2^ Nephrology, Dialysis and Transplantation Unit Careggi University Hospital Florence Italy

**Keywords:** carfilzomib, proteasome inhibitors, renal impairment, thrombotic microangiopathy

## Abstract

**Background:**

Thrombotic microangiopathy (TMA) is a pathological syndrome characterized by a combination of three key features: microangiopathic hemolytic anemia (MAHA), thrombocytopenia, and organ damage, primarily affecting the kidneys. There are several drugs known to have a definite or probable causal association with TMA, and carfilzomib, a second‐generation irreversible proteasome inhibitor (PI), approved for the treatment of multiple myeloma (MM), is one of them. In the medical literature, there have been a growing number of reports describing this serious adverse event occurring in MM patients. The precise mechanisms underlying the development of PI‐induced TMA are not yet fully understood. Significant improvements in both renal and hematological aspects have been documented following the administration of eculizumab.

**Recent Findings:**

In this report, we present two cases of MM patients who developed TMA while undergoing carfilzomib therapy. These cases were successfully treated at the Haematology Unit, Careggi Hospital in Florence. In our cases as well, the introduction of eculizumab resulted in rapid enhancements in renal function and platelet count, ultimately leading to the discontinuation of hemodialysis after 4 and 2 weeks, respectively.

**Discussion and Conclusion:**

We assessed 91 patients who received carfilzomib‐based therapies at our Haematology Department, during which we identified two cases of DITMA (2.2% incidence). Additionally, we conducted a literature review and discovered a total of 75 documented cases of carfilzomib‐induced TMA. Our experience aligns with the cases reported in literature: this adverse event can manifest at any point during treatment, regardless of the specific drug combinations used alongside carfilzomib. The initial and most crucial step in its management involves discontinuing carfilzomib therapy; therefore, recognizing TMA in a timely manner is of utmost importance. Eculizumab could play a role in improving and expediting the resolution of this potentially fatal adverse event, but further studies are needed.

In a MM patient receiving carfilzomib, presenting with anemia, thrombocytopenia, and impaired renal function, a carfilzomib‐induced TMA should be suspected in order to discontinue the causative agent.

## 1 Introduction

Thrombotic microangiopathy (TMA) is a pathological syndrome characterized by a triad of microangiopathic hemolytic anemia (MAHA), thrombocytopenia, and organ damage, predominantly affecting the kidneys [1]. It is a life‐threatening condition that requires a prompt diagnosis and treatment. TMA is classified into primary and secondary forms. Among the secondary TMAs, drug‐induced TMA (DITMA) accounts for 20%–30% of cases [2]. Among the newer therapies used for the treatment of multiple myeloma (MM), proteasome inhibitors (PIs) have been identified as causative agents in some cases of TMA. In particular, there have been increasing reports of this serious adverse event associated with carfilzomib (CFZ), a second‐generation irreversible PI. Two distinct pathogenic mechanisms have been suggested: one involves an immune‐mediated process characterized by an idiosyncratic reaction triggered by the formation of drug‐dependent antibodies, which may manifest within hours or days after the initial drug administration. The other mechanism pertains to dose‐dependent toxicity, involving various pathogenic processes such as endothelial cell dysfunction, heightened secretion of von Willebrand factor, and reduced production of prostacyclin and nitric oxide [3]. Additionally, PIs that target the ubiquitin‐proteasome pathway stabilize the nuclear factor kappa B (NFkB) complex, preventing its translocation to the nucleus. The inhibition of the NFkB pathway leads to a decrease in vascular endothelial growth factor (VEGF) production and nitric oxide concentration, resulting in diffuse endothelial damage across multiple organs [4]. Our literature analysis revealed a total of 75 documented cases of carfilzomib induced DITMA, as summarized in Table 2. Carfilzomib demonstrated an association with TMA across various pharmacological combinations, including Kd (with dexamethasone), KRd (with lenalidomide and dexamethasone), KCd (with cyclophosphamide and dexamethasone), and Dara‐Kd (with daratumumab plus dexamethasone). Furthermore, no significant difference in the incidence of DITMA cases was observed between patients who received carfilzomib in early or advanced lines of therapy (i.e., 23% of individuals receiving carfilzomib‐based therapy as a first‐line treatment) and irrespective of the treatment duration. The clinical presentation exhibited heterogeneity, with some cases manifesting only mild symptoms and malaise. Currently, in addition to drug discontinuation, there is no consensus on treatment strategies for DITMA. It appears that the complement system may play a role in the pathogenesis of this disease, supporting the potential beneficial effect of treatment with eculizumab, a monoclonal antibody against C5 [25, 26]. Herein, we describe these two cases of MM patients who were successfully treated at the Haematology and Nephrology Unit, Careggi University Hospital in Florence. We assessed 91 patients who received carfilzomib‐based therapies at our Haematology Department, during which we identified two cases of DITMA (2.2% incidence). Prompt diagnosis, immediate drug discontinuation, and early initiation of eculizumab treatment led to the discontinuation of hemodialysis and excellent recovery in patients who developed DITMA during carfilzomib therapy. In this study, we aim to emphasize that carfilzomib‐induced DITMA should be well recognized within the medical community, among not only hematologists and nephrologists, but among primary care and emergency care physicians. The diagnosis may be delayed as symptoms are often mild, and anemia, thrombocytopenia, or acute kidney injury (AKI) could be mistaken for symptoms of recently diagnosed myeloma or a relapse. Alternatively, carfilzomib‐induced TMA may be triggered by a viral infection initially considered as the cause of fever. It is crucial to promptly consider this complication in order to discontinue the causative agent and initiate supportive therapy. In particular, it is important to rapidly identify cases that may benefit most from eculizumab treatment, to avoid fatal outcomes. All patients provided written informed consent before the description of the case.

## 2 Case Reports

### 2.1 Case 1

**FIGURE 1:**
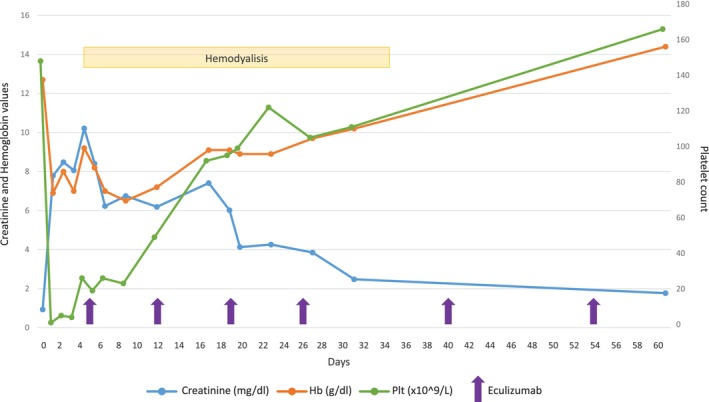
Clinical course of TMA in Case 1. The figure shows the evolution of the three main parameters (Hb in orange, creatinine in blue, and platelet count in green), before and during treatment with eculizumab (purple arrows). Progressive normalization of Hb and platelets and improvement in renal function were observed after the first four administrations, which allowed discontinuation of hemodialysis.

In February 2021, a 75‐year‐old male patient was diagnosed with IgG‐kappa MM. The patient had a history of arterial hypertension and a prior myocardial infarction. At the time of diagnosis, he presented with multiple osteolytic lesions, without anemia or AKI. Bone marrow biopsy confirmed the presence of 75% of clonal plasma cells; FISH analysis ruled out high‐risk cytogenetic alterations. He initiated treatment with KRD regimen at our hospital (carfilzomib 20 mg/smq on Day 1 of Cycle 1 and 56 mg/smq on Days 8–15 of Cycle 1 and Days 1–8–15 from Cycle 2, lenalidomide 25 mg Days 1–21, dexamethasone 20 mg weekly). After 2 days from the first full dose of 56 mg/smq carfilzomib (C1, Day 10), the patient developed fever, nausea, vomiting, and diarrhea. Three days later, given the persistence of gastrointestinal symptoms, with an episode of dark stool emission, and reduced urine output (oligo anuria); the patient went to the emergency department. At the hospital, his lab tests revealed AKI with creatinine 7.79 mg/dL (eGRF 6 mL/min vs. 105 mL/min at baseline), Grade 2 anemia (Hb 9.2 g/dL, normal range 14–18 g/dL), Grade 4 thrombocytopenia (platelet count 1 × 10^9/L, normal range 140–440 × 10^9/L), and increased lactate dehydrogenase (LDH) level (1.192 U/L, normal range 135–225 U/L). Initially diagnosed as gastroenteritis, he was treated with fluids and antibiotics. After 24 h, additional blood tests showed haptoglobin level of <0.1 g/L (normal range 0.3–2 g/L), total bilirubin of 0.5 mg/dL (normal range 0.2–1 mg/dL) and negative direct antiglobulin test (DAT). A peripheral blood smear revealed the presence of several schistocytes (seven schistocytes per field of view). ADAMTS13 activity was within the normal range (65.9%). Taken together this data were consistent with a diagnosis of a TMA, considering the simultaneous presence of the classic triad and according to the criteria proposed by Freyer et al. [18]. Upon completing the complement profile, it was noted that the patient had reduced C3 levels (0.57 g/L) and normal C4 levels (for complete results and normal ranges see Table 1). Stool cultures were negative for *Escherichia coli* O157. Autoimmune explorations for anti‐nuclear factor, anti‐DNA antibodies, ANCA, and anti‐phospholipid antibody levels all returned negative results. Kidney biopsy was not performed due to Grade 4 thrombocytopenia. After excluding other potential secondary causes of TMAs, suspicion turned to carfilzomib‐related DITMA. As renal improvement was not observed following 3 days of supportive therapy, the patient underwent hemodialysis three times a week and started eculizumab infusions. Eculizumab was administered at a dose of 900 mg weekly for the initial 4 weeks, followed by a maintenance dose of 1200 mg IV every 2 weeks. Following the firsts 4 weeks of treatment, a normalization of platelet count and a progressive improvement of renal function were reported, allowing hemodialysis discontinuation (Figure 1). Two months later, the patient resumed myeloma therapy with dexamethasone and lenalidomide (adjusted for renal function), alongside bi‐monthly eculizumab infusions. After 1 year, his kidney function improved to an eGFR of 60 mL/min, leading to the permanent discontinuation of eculizumab. Genetic testing, involving an NGS analysis of complement‐related genes (complement factor H/CFH, CFH‐related, MCP/CD46, CFB, CFI, C3, thrombomodulin/THBD), was also performed, revealing a heterozygous deletion in the CFHR3–CFHR1 region. As of today, the patient maintains good overall conditions, and it has not been necessary to resume eculizumab therapy. Renal function has remained stable over time. The patient continued myeloma treatment following the Rd regimen, achieving complete remission.

**TABLE 1:** Summary of laboratory findings at the DITMA onset in Case 1 and 2.

	Normal ranges	Patient 1	Patient 2
Creatinine (mg/dL)	(0.70–2.00)	7.79	6.4
eGFR (mL/min)	(>60)	6	9
Hemoglobin (g/dL)	(14.0–18.0)	9.2	5.4
Platelets (× 10^9/L)	(140–440)	1	5
LDH (U/L)	(135–225)	1192	1200
Total bilirubin (mg/dL)	(0.2–1.0)	1.0	2.5
Haptoglobin (g/L)	(0.30–2.00)	<0.1	<0.1
ADAMTS13 activity (%)	(50–150)	65.9	69.9
C3 (g/L)	(0.90–1.80)	0.57	0.68
C4 (g/L)	(0.10–0.40)	0.1	0.14

Abbreviations: C3, complement component 3; C4, complement component 4; eGFR, estimated glomerular filtration rate; LDH, lactate dehydrogenase.

### 2.2 Case 2

**FIGURE 2:**
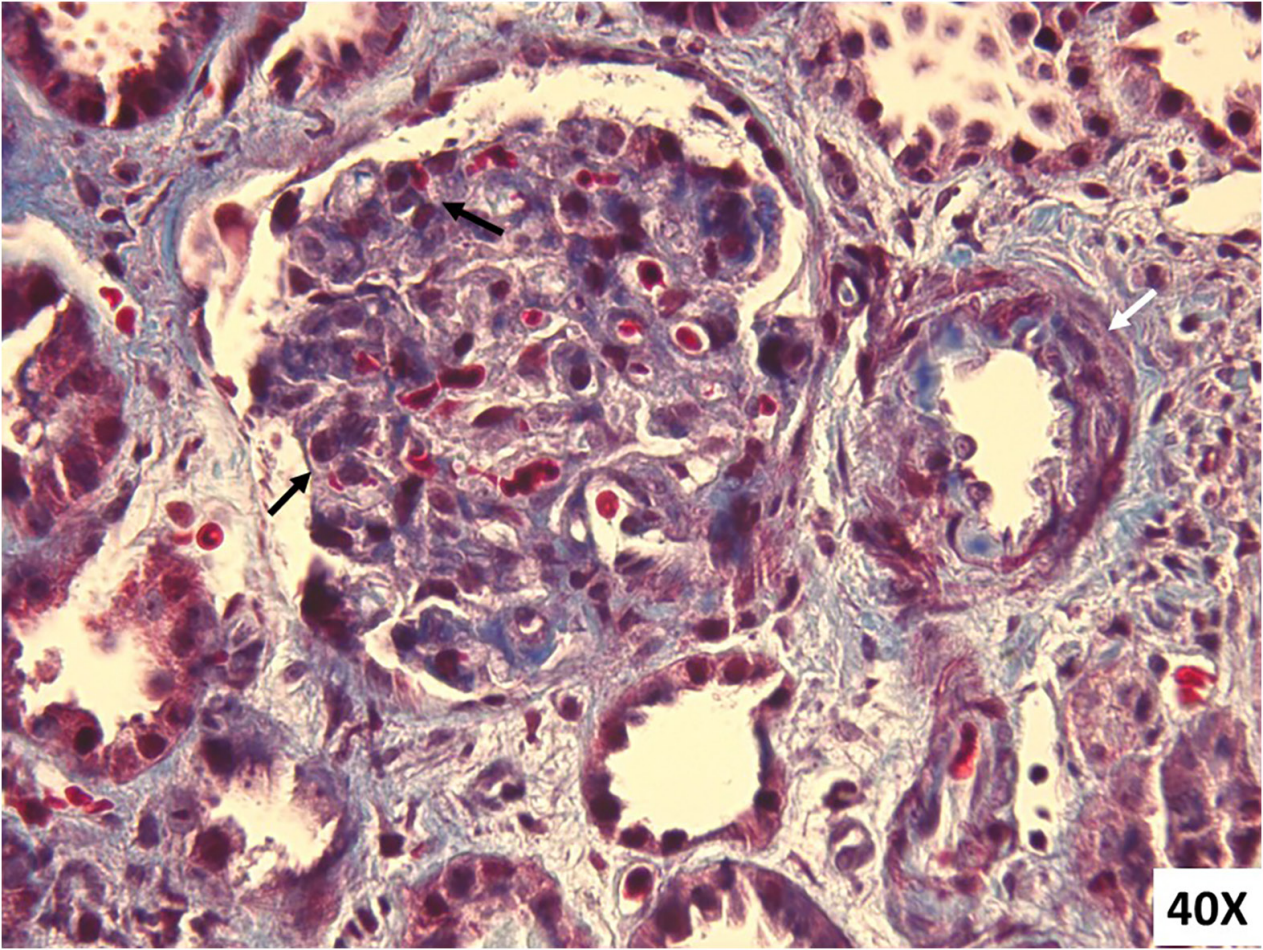
Renal biopsy findings in Case 2 showing glomerular involvement in TMA, including thickening of capillary wall due to subendothelial expansion, endothelial swelling, and fragmented red blood cells entrapped in glomerular capillaries (black arrow). Arterioles showed mild thickening (white arrow) without classical TMA associated early abnormalities (Masson trichome); [magnification 40×].

**FIGURE 3:**
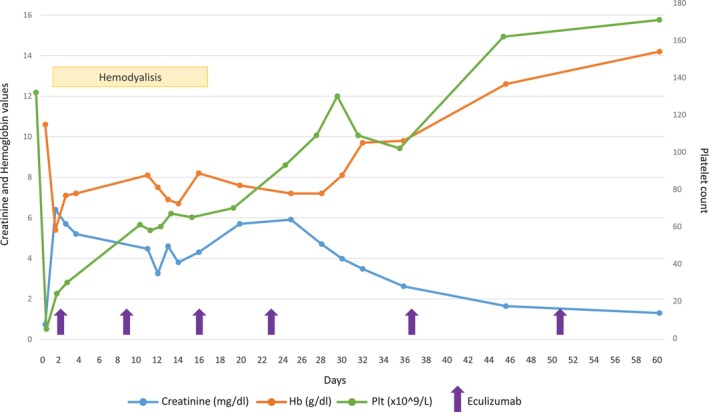
Clinical course of TMA in Case 2. Two days after the admission the patient started hemodialysis and concomitant eculizumab infusions. After 2 weeks of eculizumab treatment, there was a recovery of platelets and hemoglobin and improvement in urinary output, which allowed hemodialysis weaning off.

A 72‐year‐old woman diagnosed with IgG‐lambda MM since 2006, with a medical history of well‐controlled hypertension. She was initially treated with thalidomide and dexamethasone, followed by autologous stem cell transplantation. After 13 years, she experienced a skeletal relapse and underwent treatment with the DaraRd regimen (daratumumab, lenalidomide, and dexamethasone). In October 2022, her tests revealed a new biochemical relapse, marked by an increase in light chain levels (FLC lambda 1064.31 mg/L and FLC ratio 96). Consequently, she commenced the third line of treatment with KD regimen (carfilzomib starting dose 20 mg/smq on Days 1–2 of Cycle 1 and thereafter 56 mg/smq on Days 1–2, 8–9, 15–16, and dexamethasone 20 mg biweekly), achieving a partial response after 1 cycle. During the second cycle, the patient experienced Grade 2 arterial hypertension, leading to modifications in her antihypertensive therapy. During the third KD cycle (on Day 5), the patient reported fever, cough, and fatigue. SARS‐CoV‐2 test returned positive. Within a few days her condition deteriorated, with significant asthenia e mental confusion. She was admitted to the intensive care unit with AKI, hyperbilirubinemia, Grade 3 anemia and Grade 4 thrombocytopenia and the need for low flow oxygen support. For the first 2 days, the focus of treatment was on managing the COVID‐19 infection, but the patient's condition remained anuric, and her test results continued to worsen. She displayed anemia (5.4 g/dL), thrombocytopenia (platelet 5 × 10^9/L), fever, AKI (creatinine 6.4 mg/dL, with eGFR 9 mL/min vs. 89 mL/min at baseline), and neurological symptoms. In particular, further investigations revealed haptoglobin <0.1 g/L, LDH 1200, ADAMTS13 activity 69.9%, low C3 levels (0.68 g/L), normal C4, negative Coombs test, and multiple schistocytes in the blood smear (see Table 1 for complete results). Her lab results were consistent with a TMA. A kidney biopsy was also performed revealing focal and segmental thickening of capillary wall due to subendothelial expansion, endothelial swelling, fragmented red blood cells entrapped in glomerular capillaries along with occasional microthrombi, and moderate interstitial fibrosis (Figure 2). Immunofluorescence indicated low deposition of IgM and C3 in seven glomeruli, with no evidence of light chain deposition. Two days after her admission, hemodialysis was initiated. Dialysis was performed three times a week for 2 weeks until her diuresis improved. Simultaneously, she started eculizumab infusions, initially weekly, and later bi‐monthly, resulting in progressive improvement of renal function (eGFR 50 mL/min after 7 weeks) and normalization of platelet count (Figure 3). After 5 months of treatment, considering renal function stability and negativization of hemolysis indicators, eculizumab was interrupted. Carfilzomib was permanently discontinued. The patient is currently out of therapy and maintains close follow‐up with blood tests showing stability in response to the previous KD treatment, while renal function remains steady with GFR values hovering around 60 mL/min. An NGS panel analysis on peripheral blood was performed also in this case, revealing a heterozygous CFHR5 deletion (role in complement regulation not fully understood).

## 3 Discussion

Carfilzomib is a second generation and irreversible PI, approved for MM treatment. The increasing use of carfilzomib outside clinical trial settings, has unveiled new insights into adverse events; notably, the emergence of CFZ‐induced TMA as a rare yet potentially life‐threatening occurrence. Among 91 MM patients treated with carfilzomib‐based regimens at our center, we identified two cases of TMA, resulting in an incidence rate of 2.2%. A literature review revealed 75 reported cases of TMA associated with carfilzomib treatment (data in Table 2). Based on the reported data, the incidence of events is higher in male patients, with a median age of 60 years. The onset of TMA occurs regardless of the specific drug combination associated with carfilzomib. Additionally, the time from the initiation of therapy to the onset of TMA can vary significantly among cases. In approximately one quarter of cases (24%), such as in our Case 1, TMA developed during the initial treatment cycle, potentially indicating an immune‐mediated mechanism. Conversely, similar to Patient 2, in another 34% of patients, TMA was diagnosed between the 2nd and 11th treatment cycles. In a minority of cases (four patients), the onset occurred after prolonged exposure (14–22 cycles), suggesting a possible dose‐dependent toxicity. The clinical presentation at the onset of TMA can be highly varied, with some cases exhibiting only subtle symptoms. The most commonly reported clinical manifestations include gastrointestinal symptoms (31%), oligo‐anuria (27%), hypertensive crisis (24%), and fever (21%). Laboratory tests typically reveal the classic triad of MAHA (present in 100% of cases), thrombocytopenia (95%), and acute renal injury (98%). To rule out thrombotic thrombocytopenic purpura (TTP), it is recommended to conduct ADAMTS13 activity level testing. In our two patients and in an additional 46 cases where the test was performed, ADAMTS13 activity levels were found to be within the normal range. Complement functional activity was assessed in 27% of patients, uncovering impaired values in six individuals. The diagnosis can be histologically confirmed through renal biopsy. Literature reports on 18 patients indicate that the majority exhibit fibrin thrombi, glomerular mesangiolysis, interstitial edema, as well as areas of necrosis and fibrosis. Regarding therapy, both in our center and in the cases reported in the literature, the initial therapeutic approach typically involved prompt withdrawal of carfilzomib and the introduction of supportive therapy. In some hospitals, therapeutic plasma exchange (TPE) was initially initiated and later discontinued after ruling out a diagnosis of TTP. In our two patients, TMA initially proved refractory to carfilzomib withdrawal and plasmapheresis procedures. However, upon the introduction of the anti‐C5 monoclonal antibody eculizumab, we observed rapid improvements in renal function and platelet count. This led to the suspension of haemodialysis after 4 and 2 weeks, respectively. In several articles, significant responses, both in renal and hematological aspects, were reported following eculizumab treatment. This allowed for the discontinuation of haemodialysis in 10 out of 20 patients, within a median period of 6 weeks. The clinical benefits observed following eculizumab administration support the involvement of the terminal complement pathway in the pathogenesis of TMA. Indeed, the precise pathogenesis of PI‐induced thrombotic microangiopathies (TMAs) remains incompletely understood; however, it likely involves a multifactorial mechanism. Some known risk factors include stem cell transplantation (both autologous and allogeneic), concurrent use of causative drugs (e.g., calcineurin inhibitors), mutations in the alternative complement pathway, and infections [29].

**TABLE 2:** Summary of the literature and case reports of carfilzomib‐induced TMA.

Reference	No. of cases	CFZ dose (mg/m2)	Clinical manifestation	Creatinine increased	ADAMTS13 activity	Renal biopsy	TPE	Dialysis	Eculizumab	Outcome
Hobeika, Self, and Velez 2014 [5]	1	27	Hypertension, peripheral edema	Yes	NA	Done	No	No	No	Death due PD
Lodhi, 2015 [4]	NA	NA	Peripheral edema	Normal	Done	Yes	Yes	No	No	Recovered
Sullivan, 2015 [6]	1	NA	Oligo‐anuria, hypertension, neurological symptoms	Yes	Normal	Not done	Yes	No	No	Recovered
Qaqish, 2015 [7]	2	70	Oligo‐anuria, hypertension, gastrointestinal symptoms	Yes	Normal	Done	Yes	1 pt	No	Recovered
Chen, 2016 [8]	4	2 pts 56 2QW, 2 pts 27 2QW	Gastrointestinal symptoms, fever, oligo‐anuria	Yes	Normal	Not done	No	1 pt	No	Recovered
Yui, 2016 [9]	8	3 pts 20, 2 pts 27, 1 pt 36, 2 pts 56	Gastrointestinal and neurological symptoms	Yes	Normal	1/8 done	3 pts	4 pts	3 pts	Death due DITMA
Gosain, 2017 [10]	1	70	Hypertension	Yes	Normal	Not done	No	Yes	Yes	Recovered
Portuguese and Lipe 2018 [11]	3	27	Gastrointestinal symp, oligo‐anuria, fever	Yes	Normal	Not done	No	Yes	2 pts	Recovered
Moliz, 2019 [12]	1	56	Hypertension, fever	Yes	Normal	Not done	No	Yes	Yes	Recovered
Jindal, 2020 [13]	1	36 2QW	Gastrointestinal, fever	Yes	Normal	Done	Yes	Yes	No	Recovered
Bhutani, 2020 [14]	1	56 QW	Malaise	Yes	Normal	Not done	No	Yes	Yes	Recovered
Casiez, Pica, and Bally 2020 [15]	1	NA	Gastrointestinal symptoms, hypertension, oligo‐anuria	Yes	Normal	Not done	Yes	Yes	Yes	Recovered
Monteith, 2020 [16]	3	70 QW	Hypertension, fever	2/3	Normal	1/3 done	2 pts	No	No	Recovered
Jeyaraman, 2020 [17]	1	20 2QW	Fever, oligo‐anuria	Yes	Normal	Not done	Yes	Yes	No	Recovered
Freyer, 2021 [18]	1	20 2QW	Oligo‐anuria, neurological symptoms	Yes	Normal	Not done	No	Yes	Yes	Death due PD
Darwin, 2021 [19]	1	56 Q2W	Hypertension, neurological symptoms	Yes	Normal	Not done	Yes	No	Yes	Recovered
Rassner, 2021 [20]	2	1 pt 27 2QW, 1 pt 36 2QW	Hypertension, fever, neurological symptoms	Yes	Normal	Not done	Yes	1 pt	Yes	Recovered
Camilleri, 2021 [21]	8	56 2QW	Oligo‐anuria, hypertension, neurological symp, fever	Yes	Normal	1/8 done	7 pts	3 pts	No	Recovered
Eigbire, Hermelin and Blackall 2022 [22]	1	27 2QW	Hypertension	Yes	Normal	Not done	Yes	No	Yes	Recovered
Philipponnet, 2022 [23]	1	70	Oligo‐anuria, hypertension, peripheral edema	Yes	Normal	Done	No	Yes	Yes	Recovered
Myall, 2022 [24]	2	1 pt 56 2QW, 1 pt 70 Q2W	Gastrointestinal symp, fever	Yes	Normal	Not done	Yes	Yes	Yes	Recovered
Pallotti, 2022 [25]	1	56	Gastrointestinal sympt, fever, hypertension	Yes	Normal	Done	No	Yes	Yes	Recovered
Gavriilaki, 2022 [26]	13	10 pts 56, 3 pts 27	Unspecified	Yes	Normal	4/13 done	Yes	No	No	Recovered
Terao, 2022 [27]	5	3 pts 56 2QW, 1 pt 27 2QW, 1 Pt 36 2QW	Nausea, fever, hypertension	Yes	Normal (2/5 evaluated)	1/5 done	1 pt	2 pt	1 pt	Recovered
Moscvin, 2023 [28]	10	7 pts 56 2QW, 2 pts 56 QW, 1 pt 70 QW	Gastrointestinal symptoms, oligo‐anuria	Increased in 9/10	NA	3/10 done	5 pts	1 pt	1 pt	2 pts died due DITMA, 1 died due PD

Abbreviations: CFZ, carfilzomib; DITMA, drug‐induced thrombotic microangiopathy; NA, not available; PD, progressive disease; Q2W, every 2 weeks; QW, every week; 2QW, twice weekly; TPE, therapeutic plasma exchange.

Regarding mutations in complement genes, in both cases in our report, a heterozygous deletion in the CFHR region was identified using next‐generation sequencing (NGS) techniques. Similar findings were reported by Moscvin et al., who noted a higher frequency of deletions in the complement Factor H genes (delCFHR3‐CFHR1 and delCFHR1‐CFHR4) in MM patients with carfilzomib‐associated TMA compared to the general population and matched controls. They also identified two cases of homozygous gene deletion [28]. Gavriilaki et al. identified at least one complement‐related variant (ADAMTS13, C3, and CFH) in all TMA patient cohorts and two or more variants in 46% of them [26]. Portuguese and Lipe and Freyer et al. described three other patients with TMA and heterozygous CFHR3–CFHR1 deletions [11] [18]. Although heterozygous deletions are generally considered common benign variants in the general population, they might contribute to TMA onset in subjects susceptible to endothelial damage [30]. Regardless of complement pathway mutations, Blasco et al. demonstrated an increased deposition of C5b‐9 on endothelial cells exposed to plasma from patients with CFZ‐induced TMA, further supporting the notion that over activation of the terminal complement pathway could contribute to the pathogenesis of TMA. Knowledge of complement status might be useful in identifying patients who could benefit from terminal complement blockade [31]. The definitive treatment regimen and its optimal duration are not yet standardized. Olson et al. suggest eculizumab administration in doses typically used for atypical haemolytic uremic syndrome (aHUS) (900 mg IV weekly for 4 weeks during the induction phase, followed by maintenance with 1200 mg IV every 2 weeks). They also recommend attempting eculizumab discontinuation only after achieving hematologic recovery, with subsequent careful monitoring of laboratory tests [32]. Among cases reported in the literature, almost half of the patients treated with eculizumab discontinued treatment after achieving hematological recovery and improvement in kidney function [11, 12, 14, 15, 20, 24]. Portuguese and Lipe and Moliz et al. administered eculizumab for up to three weekly doses, while Myall used it for only two weekly doses [11, 12, 24]. In contrast, Bhutani et al. and Rassner et al. continued eculizumab treatment for 3 and 2 months, respectively [14, 20]. Regarding our two patients, both successfully discontinued eculizumab. In particular, the first patient continued treatment for a year until renal function stabilized. Based on this experience, the patient in Case 2 discontinued eculizumab after 5 months, once kidney function normalized and without any signs of haemolysis. Furthermore, it is essential to consider other concomitant triggers of TMA during carfilzomib treatment, such as the concurrent use of drugs causally associated with DITMA, intercurrent viral infections, or uncontrolled hypertension. In the case of our patient in Case 2, a concurrent SARS‐CoV‐2 infection was present, similar to six other cases reported in the literature [8, 11, 20, 23, 24]. Infectious stimuli, especially of viral origin, have been suspected as potential triggers for carfilzomib‐induced TMA.

## 4 Conclusion

Carfilzomib‐induced TMA is a rare complication in patients with MM, occurring in approximately 2% of cases in our experience. As data from the literature show, it is difficult to predict its onset and identify patients at greater risk of developing it. However, it can lead to significant comorbidities and, in some cases, be fatal. With our article, we aim to raise awareness of this increasingly important adverse event, which should be known not only by hematologists and nephrologists, but also by primary care and emergency room physicians, who are often the first to assess patients in emergency situations. In a patient with MM receiving carfilzomib who presents with anemia, thrombocytopenia, and impaired renal function, carfilzomib‐induced TMA should be suspected and differentiated from other potential causes. It is essential to promptly consider this complication to discontinue the causative agent and initiate supportive therapy. In particular, it is important to quickly identify cases that could benefit most from treatment with eculizumab to avoid fatal outcomes.

## Author Contributions

I.A. was responsible of the study design, collected the clinical data, and wrote the report. L.P. and G.T. collected the data. S.P. and I.A. was responsible for the analysis and interpretation data. L.C. was responsible for collection data about kidney biopsy. M.M. was responsible for the review of the bibliography. A.M.V. and E.A. contributed to the critical review and finalised the report. All authors have read and agreed to the published version of the manuscript.

## Conflicts of Interest

The authors declare no conflicts of interest.
